# Integrating pore architectures to evaluate vascularization efficacy in silicate-based bioceramic scaffolds

**DOI:** 10.1093/rb/rbab077

**Published:** 2021-12-16

**Authors:** Fanghui Wu, Jun Yang, Xiurong Ke, Shuo Ye, Zhaonan Bao, Xianyan Yang, Cheng Zhong, Miaoda Shen, Sanzhong Xu, Lei Zhang, Zhongru Gou, Guojing Yang

**Affiliations:** 1 Department of Orthopaedics, The Third Hospital Affiliated to Wenzhou Medical University & Rui’an People’s Hospital, Rui’an 325200, China; 2 Bio-nanomaterials and Regenerative Medicine Research Division, Zhejiang-California International Nanosystem Institute, Zhejiang University, Hangzhou 310058, China; 3 Department of Orthopaedics, The First Affiliated Hospital, School of Medicine of Zhejiang University, Hangzhou 310003, China

**Keywords:** pore geometry, vascularization, precise manufacturing, integrating pore architectures, digital light processing

## Abstract

Pore architecture in bioceramic scaffolds plays an important role in facilitating vascularization efficiency during bone repair or orbital reconstruction. Many investigations have explored this relationship but lack integrating pore architectural features in a scaffold, hindering optimization of architectural parameters (geometry, size and curvature) to improve vascularization and consequently clinical outcomes. To address this challenge, we have developed an integrating design strategy to fabricate different pore architectures (cube, gyroid and hexagon) with different pore dimensions (∼350, 500 and 650 μm) in the silicate-based bioceramic scaffolds via digital light processing technique. The sintered scaffolds maintained high-fidelity pore architectures similar to the printing model. The hexagon- and gyroid-pore scaffolds exhibited the highest and lowest compressive strength (from 15 to 55 MPa), respectively, but the cube-pore scaffolds showed appreciable elastic modulus. Moreover, the gyroid-pore architecture contributed on a faster ion dissolution and mass decay *in vitro*. It is interesting that both μCT and histological analyses indicate vascularization efficiency was challenged even in the 650-μm pore region of hexagon-pore scaffolds within 2 weeks in rabbit models, but the gyroid-pore constructs indicated appreciable blood vessel networks even in the 350-μm pore region at 2 weeks and high-density blood vessels were uniformly invaded in the 500- and 650-μm pore at 4 weeks. Angiogenesis was facilitated in the cube-pore scaffolds in comparison with the hexagon-pore ones within 4 weeks. These studies demonstrate that the continuous pore wall curvature feature in gyroid-pore architecture is an important implication for biodegradation, vascular cell migration and vessel ingrowth in porous bioceramic scaffolds.

## Introduction

Osteoporotic fracture, tumor resection and other life-threatening conditions may lead to some bone defects or irreversible bone damages, and these clinical problems remain much challenge due to its particularly pathological conditions or repair difficulties [1, 2]. In the treatment of severe oculo-orbital traumas or intraocular malignancies, it is sometimes necessary to surgically insert an orbital implant into the anophthalmic socket for volume replacement and restoring the esthetic appearance of a normal eye. In general, the prerequisite for bone repair is vascularization in the porous scaffolds, which could enhance bone regeneration and repair [3, 4]. Rapid angiogenesis during orbital reconstruction may avoid infectional exposure of porous orbital implant. Infections lead to the failure of orbital implants, which necessitates implant revision surgery. However, most artificial biomaterials used clinically including polymers, Ca-phosphate ceramics or metal implants exhibit slow vascularization due to insert surface or poor bioactivity [5–8]. Ideally, the implants should not only have a desirable porous structure with patients-tailored shapes, but also be high vascularization and mechanically strong with favorable structural stability.

Over the last two decades, the implant design and the criteria of candidate selection evolved from metal and polymer to bioactive ceramic devices with complex internal structure and functionalities for ensuring better clinical outcomes in the early time stage [9–11]. Ca-silicate bioceramic implants have gained prominence since their high bioactivity and their interconnected pore architectures would allow them to act as a passive framework for fibrovascular ingrowth [12–14]. However, some critical aspects of today’s porous implants include the structural stability and biological performances. Hence, the development of new silicate-based scaffolds with finely tuned pore architecture and enhanced host responses (e.g. angiogenesis and integration), which enable an improved outcome of bone repair or eye replacement is more than ever desirable long-term outcome.

Pore architecture, a classic topic in tissue-engineering scaffolds because of their significance in determining the structural stability and tissue ingrowth efficiency, have always attracted much attention in regenerative medicine from 1990s [15–17]. In general, the pore interconnection is necessary because these structure features determine the efficiency of nutrient transport, cell migration and vessel formation, and even the mechanical stability of porous architectures [18, 19]. Many researchers have design the bioceramic scaffolds with increasing structural complexity, and more and more studies have recognized that the other pore architecture features, such as pore geometry and pore wall curvature are also the potential factors to influence the cell migration and tissue regeneration efficiency. In fact, many concepts have been developed to understand the influence of pore size and geometry characteristics on the neovascularization of engineering high-precision pore networks [20–22]. In this context, it is important to understand the mechanical and biological properties of the porous scaffolds with different curvature pore surface and pore geometries as reported previously [23–25]. However, it is still difficult to precisely tune pore structures of scaffolds by conventional template replica or extrusion-based 3D printing techniques [10, 26]. Moreover, the existing findings involving the early bone regeneration or blood vessel ingrowth in porous scaffolds could not offer a versatile guide to develop scaffolds for clinical translation.

In this study, we aimed to fabricate the bioceramic scaffolds with three types of pore geometries and to investigate the effect of pore architecture features (pore size, pore wall surface curvature and strut corner) on the mechanical properties, ion release, bio-dissolution *in vitro* and vascularization in the early stage *in vivo*. In particular, the different pore sizes were integrated into a single scaffold and the vascularization efficacy *in vivo* was evaluated systematically. It is well known that digital light processing (DLP) strategy is versatile for preparing the porous constructs with non-conventional pore curvature (e.g. triply periodic minimal surfaces) and complex shapes via computer-aided design and computer-aided manufacturing [27, 28]. Therefore, we compared the early-stage vascular behavior of scaffolds with the strut-based pore architecture (i.e. cube or hexagon pore) and curve pore architecture (gyroid pore). The structural parameters including porosity, pore dimension and specific surface area were characterized by computer-assisted calculation. To our knowledge, our works demonstrate for the first time a proof of concept for using a pore geometry and size strategy to accelerate vascularization in the porous bioceramics.

## Materials and methods

### Chemicals and materials

The photo-sensitive resin was brought from Ten Dimensions Technology Co., China. The reagent-grade inorganic salts and trishydroxymethylaminomethane (Tris) were bought from Sinopharm Reagent Co., Shanghai, and used directly. Tris was also used to prepare to the 0.05 M Tris buffer (pH ∼7.25).

### Preparation of CSi-Mg8 ceramic powders

The pure CSi-Mg8 powders were synthesized by a wet chemical co-precipitation method as reported previously [29]. The obtained white precipitate was dried at 120°C for 24 h and then calcined at 880°C for 2 h. Finally, the as-calcined powders were ground in ethanol medium for 6 h by a planetary ball miller (Chishun Sci&Tech Co., China) to get superfine powders particle size below 8 μm.

### Fabrication of porous scaffolds by DLP technique

**Figure 1.**
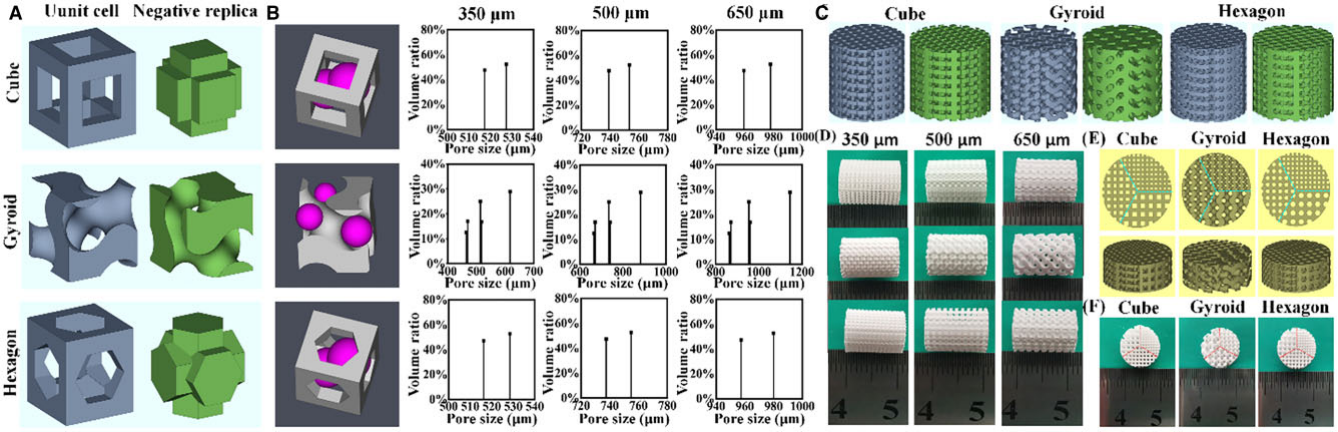
Primary characterization of the 3D models and bioceramic scaffolds. (**A**) Three types of unit cell model (cube, gyroid and hexagon) and negative replicas of pore region (green). (**B**) 3D definition of the pore size via the maximum diameter of a virtual microsphere fitting within the pore architecture at any given point within the pore space and the pore size distribution of the pore architecture in unit cell of 3D models with three types of pore geometries. (**C**) 3D models of the bioceramic scaffold with different pore geometries and pore dimensions and negative replicas of pore region (green). (**D**) Outward appearance of the representative as-sintered bioceramic scaffolds with different pore dimensions. (**E**) Top-view and 3D models of bioceramic scaffold with integrated 350-, 500- and 650-μm pore dimension. (**F**) Outward appearance of the as-sintered scaffolds with integrated 350-, 500- and 650-μm pore dimension.

The porous scaffold models were designed through 3D periodic filling the unit cells (cube, hexagon and gyroid) by the software of Materialise Magics 21.0 (Fig. 1A). The unit cell models of cube and hexagon were designed with consistent pore size and porosity by Magics 21.0, while the software of Mathmod was used to create the unit cell model of gyroid (see Supplementary Fig. S1) [30]. The designed porosity of three types of scaffold was ∼58%. As for the pore dimensions, the ∼350, 500 and 650 μm in pore dimension were selected as the benchmark, and the software of Avizo was used to calculate the pore size distribution of unit cell models in 3D definition for ensuring that the average pore dimensions of gyroid- and hexagon-pore scaffolds were the same as cube-pore scaffolds through changing the size of the unit cells (Fig. 1B) [31]. The scaffold models with dimensions of Ø 10 × 10 mm were designed for evaluation *in vitro* (Fig. 1C), and the scaffold samples with different pore geometries (cube, gyroid and hexagon) and pore dimensions (∼350, 500 and 650 μm) were signed as cube-350, cube-500, cube-650, gyroid-350, gyroid-500, gyroid-650, hexagon-350, hexagon-500 and hexagon-650, respectively (Fig. 1D). Moreover, the different pore dimension-integrated scaffold models designed as Ø 10 × 4 mm were used for animal test *in vivo* (Fig. 1E and F).

The bioceramic slurry was prepared through high-speed mechanical stirring of 60 wt% CSi-Mg8 powders and 40 wt% photo-sensitive resins. Then, the scaffold model designed previously was sliced to .STL file at 25 μm per layer and imported into the DLP printer (Ten Dimensions Technology Co., Beijing). In order to avoid the over high linear shrinkage of the sintered scaffolds, the 3D models were enlarged by 1.33-fold before printing, and the printing parameters were adjusted to reach the expected layer thickness. The printed samples were formed by layer accumulation from pattern of ultraviolet light (405 nm) according to the digital file. The obtained samples were washed ultrasonically in deionized water to remove excess slurry and followed dried at 60°C for 24 h. Finally, the porous samples were sintered at a target temperature of 1150°C with a heating rate of 2°C/min for 2 h (maintaining 1 h at 400°C to volatilize resin completely) in a muffle furnace, and then allowed to cool down naturally.

### Morphology, microstructure, mechanical analysis and finite element analysis

The outward morphology of the sintered scaffolds was observed by mobile camera (P30, Huawei). The top- and side-view pore architectures of scaffolds were observed by scanning electron microscopy (SEM, GEMINI 300, Germany). The average pore size (two parallel edges in the top-view) of porous scaffolds was measured and 12 pores from three samples were used for statistical analysis. The real scaffold porosity (*n *=* *8) was calculated via the following equation:

Real porosity=(V−m/ρ)/V×100%,

where *V*, *m* and *ρ* represent the actual volume, mass of the porous scaffolds and theoretical density of wollastonite, respectively. The cylindrical samples (Ø 10 × 10 mm, *n *=* *8) were prepared for compressive strength and elastic modulus measurements. Mechanical testing was performed using a universal mechanical test machine at a crosshead speed of 0.5 mm/min according to the procedure described in ASTM C773-88. The elastic modulus was calculated through the linear region of the stress–strain curves. In order to understand the mechanical behavior of different unit cells with three types of pore geometries under pressure, the stress distribution of the 1 × 1 × 1 unit cell under a compression of 20 N was revealed through the finite element analysis function of ANSYS software.

### Biodegradation *in vitro* evaluation

For evaluation of the bio-dissolution (biodegradation *in vitro*) of bioceramic scaffolds, the weighed porous samples (m_0_; Ø 10 × 10 mm, *n *=* *6) with different pore geometries and pore dimensions were immersed in Tris buffer (pH 7.4) with an L/S ratio of 50 ml/g and shaking in water-bath at 37°C. The 20% volume of solution was refreshed every 7 days. At the pre-set soaking time intervals, 1.0 ml of supernatant extracted from solution was mixed with 9 ml of deionized water and the inorganic ion concentrations were measured by inductively coupled plasma (ICP; Thermo, USA). After immersion for every 2 weeks, the samples were washed with absolute ethanol, dried at 80°C and then weighed (m_*t*_). The mass decrease of porous scaffolds was calculated as follows: Mass loss = m_*t*__/_m_0_ × 100%.

### Surgical procedure of implantation

In order to investigate the effects of the scaffolds with different pore structures and sizes on vascularization *in vivo*, the dorsal muscle embedding model in New Zealand rabbits was used. All animal experiments were performed in accordance with the Animals Ethics Committee of Zhejiang University (2021-0228003). Sixteen male New Zealand white rabbits (∼2.8 kg) were randomly divided into two groups, and six of them were performed by angiography before euthanasia. All rabbits with free access to food and water were allowed to adapt for 2 weeks in stainless steel cages before surgery. The rabbits were anesthetized with general intravenous injection of 3% sodium phenobarbital (Merck, Germany) at 1.0 mg/kg and fixed on the operating table for shaving and disinfection of surgical regions. Under aseptic conditions, three 1.5-cm longitudinal skin incisions were made on both sides of the spine of each animal in the back. Then six small pockets were formed by blunt dissection of muscles under the fascia. The different pore dimension-integrated scaffolds (Ø 10 × 4 mm) were implanted into pockets randomly (Fig. 5A) and the wounds were sutured layer by layer [32]. Postoperatively, penicillin was given once daily for 3 days. At 2 and 4 weeks, the rabbits were euthanized and the implants were harvested for histological evaluation of blood vessels.

### Micro-angiography and Micro-CT reconstruction

Six rabbits underwent micro-angiography at 2 and 4 weeks after operation. Under general and deep anesthesia, the thoracic cavity was opened. The aorta was cannulated with an 18-gauge venula needle and flushed with heparinized saline, then, the vena cava was severed for an outlet. After limpidity of the fluid from vena cava, contrast agent (Microfil MV-122 [yellow]; Flow Tech, USA) was injected in the aorta according to the manufacturer’s protocol (Fig. 5B) [33]. The animals were then euthanasia and stored at 4°C overnight. In the second day, the implants were harvested, fixed with 4% paraformaldehyde solution for 3 days. Micro-CT scanner (μCT; Inveon, Siemens, Germany) was used for the assessment of blood vessels volume in specimens. The colorful images of the implants were reconstructed with standardized thresholds for further quantitative analysis of the blood vessel volume/total volume (BV/TV) ratio by Inveon Acquisition Workplace (IAW, Siemens, Germany).

### Histological analysis

All implants without micro-angiography were fixed in 4% paraformaldehyde solution for 3 days, dehydrated with successive alcohol concentrations (80–100%) and cleared with xylene, then embedded in polymethylmethacrylate. The sections with thickness of ∼20 μm were cut, and then stained with hematoxylin/eosin (H&E) for angiogenesis observation using light microscope (DM500, Leica, Germany). For histomorphometric analysis, five sections of the central part from each sample were observed and then measured quantitatively using ImageJ software. The area percentage of blood vessels was calculated by the total area of blood vessels against the area of section. For each blood vessel, only the minor axis was regarded as the diameter of blood vessel [20].

### Statistical analysis

All data were presented as mean ± standard deviation. Multigroup comparisons of the means were carried out by analysis of variance tests with *post hoc* contrasts by Bonferroni test using SPSS 21.0 (IBM, USA). All results were considered to be statistically significant when *P *<* *0.05.

## Results

### Macroscopic observation of the bioceramic scaffolds

The phase compositions of the calcined CSi-Mg8 powder and sintered scaffolds have been measured by XRD analysis and both were the low-temperature β phase of wollastonite (β-CaSiO3), free of any other secondary phase (not shown). In order to allow good permeability favorable for cell migration and nutrition transport, the theoretical porosity of bioceramic scaffolds was 58% and three types of pore size (∼350, 500 and 650 μm) were designed in the cube-pore scaffolds. The pore dimension of the other two types of hexagon- and gyroid-pore scaffolds were calculated by the pore volume of the unit cell of cube pore (Fig. 1A). Therefore, the measured pore sizes of sintered cube-pore scaffolds (Table 1) were very matched with the designed pore sizes (from 350 to 650 μm) due to pre-enlargement (∼33%) of the 3D model dimensions before printing. However, the gyroid- and hexagon-pore scaffolds showed slightly higher pore size (two parallel edges from the top-view) of porous scaffolds. It is worth mentioning that, meanwhile, the measured porosity (∼55.1–56.5%) was similar to the theoretical value (∼58%) in all of the scaffold models. The pore size distribution of modeled porous constructs showed slightly different among the three types of scaffolds, which was mainly attributed to the different pore wall features and interconnected pore dimensions (Fig. 1B and C). The scaffolds appeared mechanically rigid to naked eyes. It was worth noting that the linear shrinkage of the three types of scaffolds with different pore geometries (cube, hexagon and gyroid) were similar to each other (∼23.4–24.8%; *P *<* *0.05), and there was no other non-linear deformation during undergoing process (Fig. 1D). In order to effective evaluate the effect of the pore dimensions on vascularization efficacy of three types of bioceramic scaffolds, the 350-, 500- and 650-μm pore networks were integrated into the same scaffold, and the macroscopic observation also confirmed the feasibility of the DLP-based 3D printing technique (Fig. 1E and F).

**Table 1.** Geometrical parameters of the porous scaffolds

Geometrical parameters	Cube			Gyroid			Hexagon		
	350 μm	500 μm	650 μm	350 μm	500 μm	650 μm	350 μm	500 μm	650 μm
Theoretical pore size (μm)	350	500	650	410	560	720	380	540	700
The measured pore size^a^ (μm)	344.6 ± 4.6	478.2 ± 7.6	625.8 ± 9.3	376.4 ± 6.3	551.7 ± 9.2	691.2 ± 8.9	368.4 ± 5.1	524.6 ± 7.8	682.1 ± 9.7
Theoretical porosity (%)	58	58	58	58	58	58	58	58	58
The measured porosity (%)	55.9 ± 0.6	56.3 ± 0.3	56.4 ± 0.4	56.3 ± 0.4	55.7 ± 0.3	56.5 ± 0.5	55.1 ± 0.7	55.8 ± 0.4	55.4 ± 0.3
Specific surface area (m^−1^)	12.11	8.80	6.96	13.60	9.64	7.53	11.18	8.16	6.47

^a^ The measured pore size (two parallel edges in the top-view) was obtained by statistical analysis from SEM images.

### Structural characterization of bioceramic scaffolds

**Figure 2.**
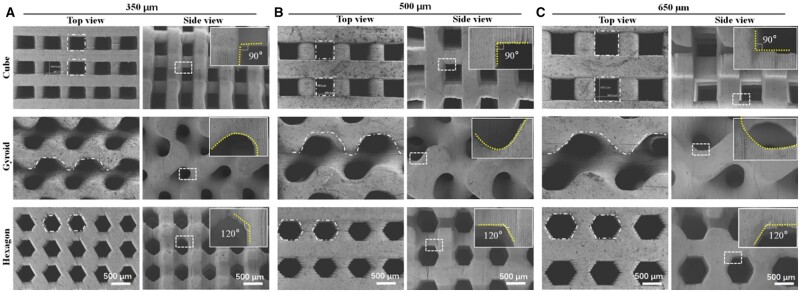
SEM images of the top-view and side-view surface morphology of bioceramic scaffolds with different pore geometries. The dotted lines and frameworks in top-view images confirming the pore shape, and insets in side-view images showing the magnification of the pore morphology.

The bioceramic scaffolds were printed via a protocol developed in our group as reported previously [16, 24]. After sintering at 1150°C, the pore morphology and microstructures of the scaffolds were observed under SEM. As shown in Fig. 2, the SEM images from the top- and side-view of scaffolds demonstrated that the pore geometries were fully consistent with the designed pore models, with no noticeable defect or deformation of the unit cell. The pore wall displayed similar microstructure and densification, which was mainly attributed to their identical chemical composition and sintering condition. The cubic pore has been widely reported in document, and it represents the conventional pore morphology in 3D printed bioceramic scaffolds [34, 35]. The SEM observation confirmed that the strut size was precisely controlled consistently in the DLP-based 3D printing bioceramic scaffolds, and the pore size was increased with the increase of length of the side from 350 to 650 μm in the 3D models. As expected, the hexagon pore maintained the six-side structures and the angle between adjacent edge was ∼120° after sintering, which is consistent with the 2D model of the hexagon pore. Meanwhile, the gyroid-pore structure showed curve surface with similar curvature, regardless of the difference in pore dimensions. Totally, it was confirmed the flexibilities of printing bioceramic scaffolds with various internal pore architectures, and the pore dimensions of scaffolds with precisely defined pore geometries could be readily controlled after sintering.

### Mechanical evaluation of bioceramic scaffolds

**Figure 3.**
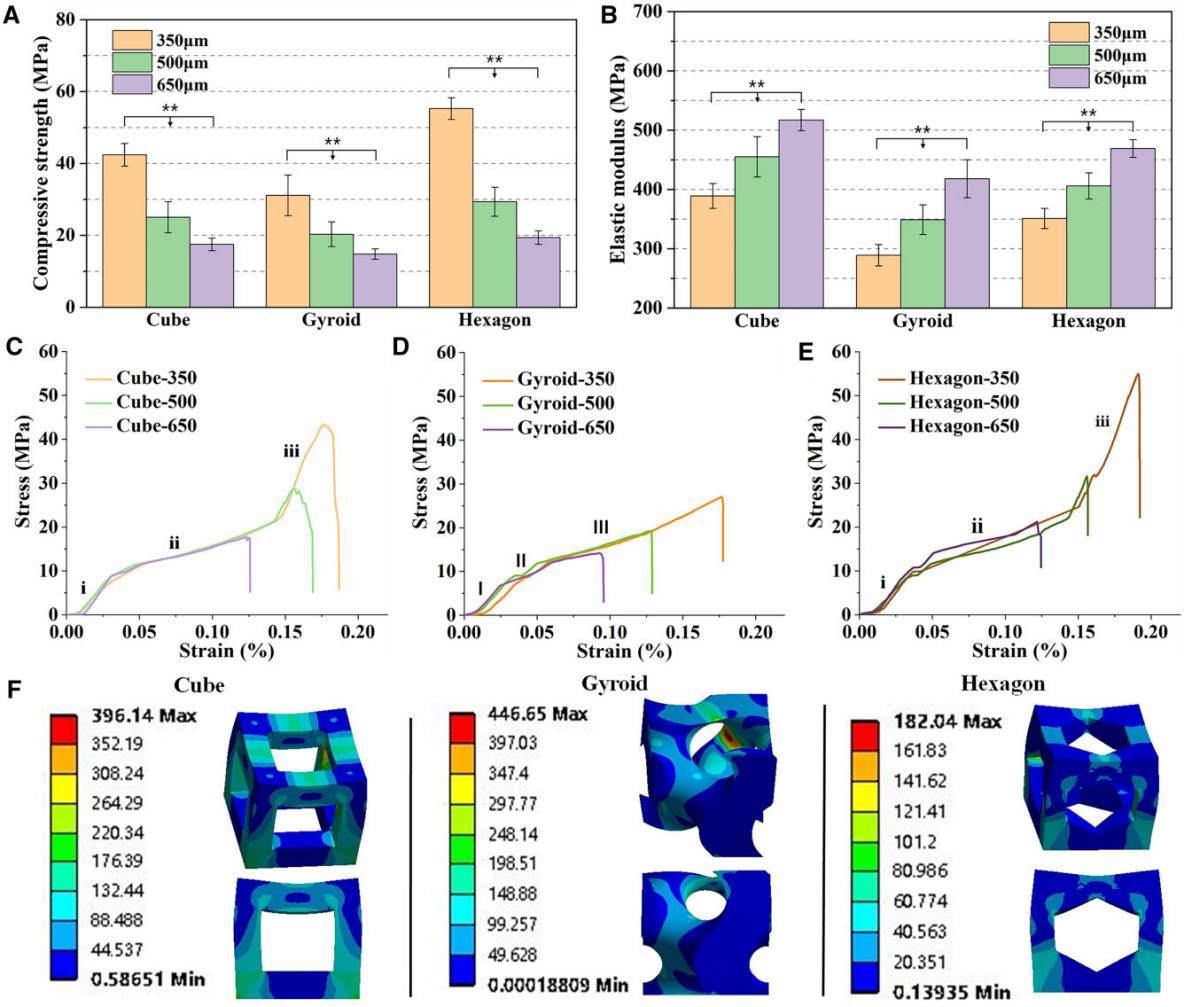
Compressive strength (**A**), elastic modulus (**B**) and representative stress–strain curves (**C–E**) of the porous bioceramic scaffolds. (**F**) Stress distribution of the three types of unit cell (cube, gyroid and hexagon) under compression load (**P < 0.01).

Figure 3A shows the compressive strength of bioceramic scaffolds with different pore geometries and pore dimensions. It was evident that the scaffolds with low pore dimension exhibited higher compressive resistance. The 350-μm hexagon-pore scaffolds showed highest compressive strength (∼55 MPa) and the gyroid-pore scaffolds with similar pore dimension had lowest strength (∼30 MPa). With the increase of pore dimension, the mechanical strength was decreased among three types of scaffolds, which was consistent with previous studies [19]. Especially, the bioceramic scaffolds from 350 to 500 μm showed a more significant decrease in strength decay (∼33% vs 47%), and this may be related to higher base value. On the other hand, the elastic modulus was increased with the increase of pore dimension (Fig. 3B). The cube-pore scaffolds exhibited the higher values than the other two types of scaffolds with similar pore dimension. This can be elucidated that the pore struts along the *Z* axis are parallel to the compressive force orientation in the cube-pore scaffolds so that the fracture surface of pore struts may undergo a higher compression resistance before deformation or structure collapse.

Figure 3C shows the representative stress–strain curves for bioceramic samples under compression. It is obvious that the stress–strain graphs followed a similar trend as typical brittle ceramics, but the gyroid-pore scaffolds showed a slight difference in elastic responses in comparison with the other types of scaffolds. As for the gyroid-pore scaffolds, the stress was increased slowly (region I; ∼5% in strain), and then there occurred yield a minor terrace in the stress–strain curves (region II) for the samples, which corresponded to the yield strength at the end of the elastic deformation. After that, the stress was linearly increased during which the pore walls continued to densifying progressively (region III), an effect known as densification process. However, it can be seen from the stress–strain graphs of cube- and hexagon-pore scaffolds that the stress was increased approximately linearly with deformation in the initial two regions (region i1 and i2; accompanying with ∼3% and ∼10% strain), implying the elastic resistance of the porous scaffolds. However, it is interesting that the intensity and sharpness of the peaks decreased with an increase of pore dimension of scaffolds, and the stress for the 350-μm pore scaffolds continued to increase significantly (region i3). It may be postulated that the broken pore walls were compacted until they reached the peak of compressive resistance at which point all of pore wall broke.

The finite element analysis was used to understand the stress distribution of the three types of unit cell under stress condition (Fig. 3F). The color of blue to red indicated the low to high stress intensity. The unit cells of gyroid and hexagon showed the highest (446.65 Mpa) and the lowest (182.04 Mpa) stress maximum, respectively. It was evident that hexagon-pore structure exhibited a more homogeneous stress transport. However, the gyroid structure possessed a strong stress concentration point, which may easily lead to structural fracture.

### Bio-dissolution behavior *in vitro*

**Figure 4.**
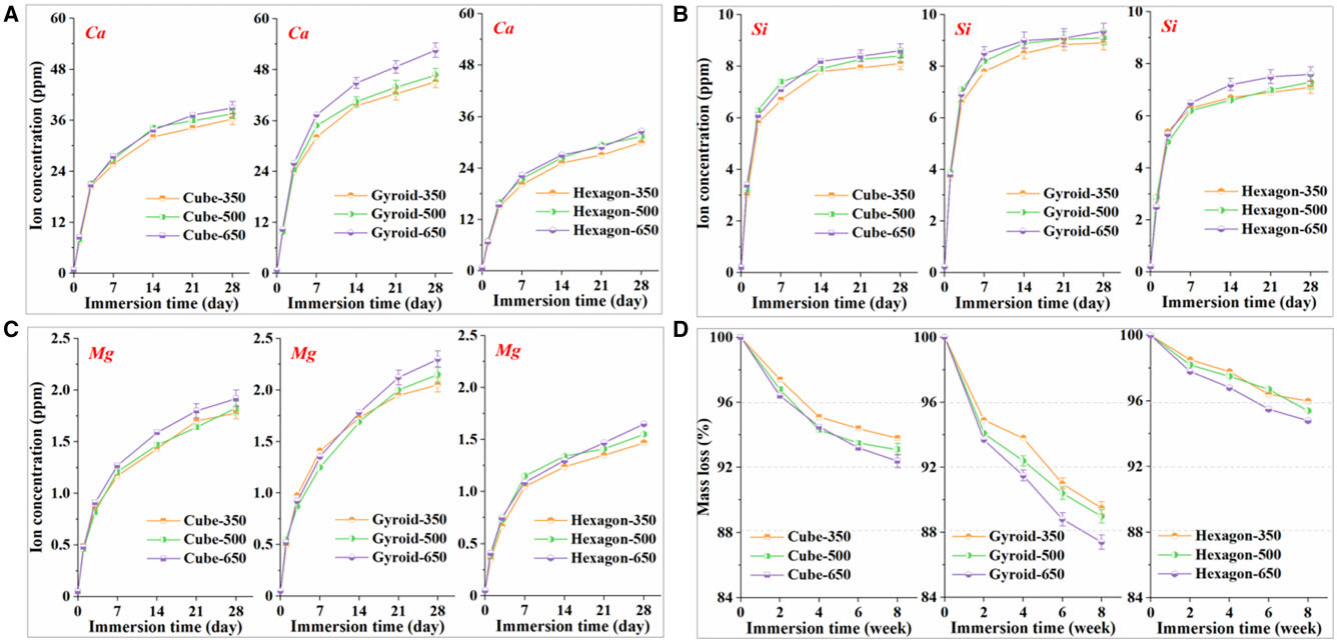
Changes in Ca (**A**), Si (**B**) and Mg (**C**) concentrations in tris buffer (pH7.4) during immersing the scaffolds for 28 days. (**D**) Mass decrease of bioceramic scaffolds during immersion in tris buffers for 8 weeks.

The bio-dissolution test for bioceramic samples was carried out in Tris buffer *in vitro* and the ion release behavior was evaluated for a long time stage (28 days). Figure 4A–C shows that the concentrations of Ca, Si and Mg were increased quickly within the early stage of 7 days, and then a slow increase was maintained up to 28 days. However, a significant pore geometry-dependent increase in the ion dosage was observed. It is indicated that the gyroid- and hexagon-pore scaffolds showed respectively the fastest and slowest ion release in the whole immersion stage. A trend toward an increasing Ca concentration in the gyroid-pore scaffold group was noticed but the differences between groups with different pore dimensions were not statistically significant (*P* > 0.05). Out of these, two types of metal ions (Ca^2+^, Mg^2+^) were of interest to us: Ca^2+^ present as the main cations in the Ca-silicate compound and Mg^2+^ present as the doped ions, which partially substitute Ca (8% Ca^2+^ was substituted by Mg^2+^). Accumulation of Mg ions in the buffer solution was due to bio-dissolution of non-stoichiometric wollastonite ceramic into the aqueous medium. We observed a stable value in the ratio of Ca/Mg (∼23:2) with an increase of immersion time indicating a continuous dissolution of the bioceramic scaffolds with an increase of immersion time.

Simultaneously, the scaffolds were further assessed for mass decay in Tris buffer for a long time stage (8 weeks). The degree of mass loss in the buffer solutions was quantified directly through weighing the dried samples. Both pore geometry- and dimension-dependent increase were observed in the whole time stage (Fig. 4D). The hexagon-pore architecture could contribute on higher structural stability, and the mass loss of this type of scaffolds was only ∼7% after immersion in for 8 weeks. However, the gyroid-pore scaffolds showed significantly fast mass decrease (∼4.7–6.3%) within 2 weeks, and only 88–90% mass was retained after 8 weeks.

The ion release and mass loss of the porous constructs with different pore geometries at each time point were possibly attributed to the significantly higher specific surface area (Table 1) of the three types of unit cell. It was noted that, although the gyroid-pore scaffolds had lower specific surface area with increasing the pore dimension, the ion release and mass loss were faster in the large pore scaffolds. This was probably attributed to a fact that the larger is the pore dimension, the faster is the ion exchange. That is, the large pore in bioceramic scaffolds may be beneficial for enhancing the biodegradation rate *in vitro*. Moreover, immersion of the scaffolds in Tris buffer for 8 weeks did not alter their surface morphology and pore geometries as can be seen to naked eye.

### Primary evaluation for the dorsal muscle embedding models

**Figure 5.**
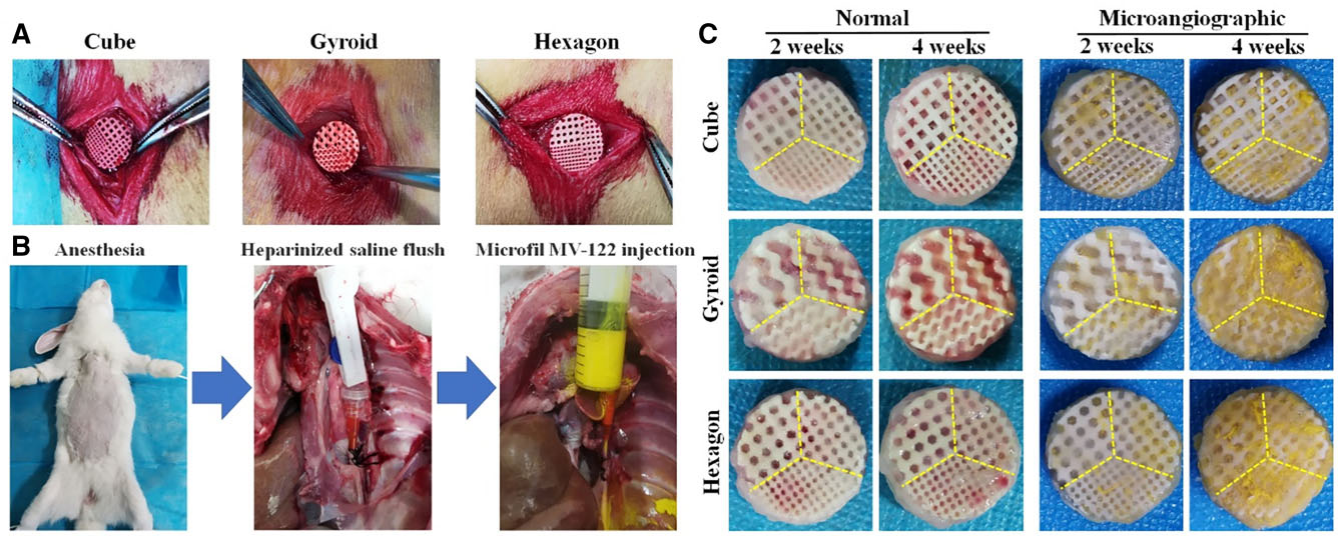
(**A**) Implantation of the different pore dimension-integrated scaffolds in dorsal muscle. (**B**) Schematic illustration of micro-angiography in rabbits after operation at 2 and 4 weeks. (**C**) Normal and micro-angiographic implants harvested from rabbits after implantation for 2 and 4 weeks.

Since the pore geometry and surface feature of the scaffolds with three different pore dimensions were significantly different but the pore structural parameters had negligible difference (Table 1), we decided to integrate the different pore dimensions into a single scaffold for angiogenesis analysis of the dorsal muscle embedding model *in vivo*. The animal model experiments involving the implantation of bioceramic scaffolds beneath the dorsal muscle in the back of rabbits (Fig. 5A) were carried under the standard animal care condition. The porous implants were fabricated by integrating 350-, 500- and 650-μm pore dimensions into the same scaffold via DLP-based 3D printing. All of rabbits were awake at 1 h postoperatively. Figure 5B displays the injection operation with contrast agent after 2 and 4 weeks of scaffold implantation. Figure 5C shows the representative outward appearance of bioceramic scaffolds specimens without and with injection of contact agent, respectively. It was noted that no rabbits showed obvious infection, and all of them were survived long enough until the different time points (2 and 4 weeks) for material harvest. In fact, the gross examination of the porous samples at 2 and 4 weeks (Fig. 5C) showed that the new blood vessels were migrated into the pore networks and no inflammatory response was observed in all of specimens.

### μCT-based angiographic reconstruction analyses

**Figure 6.**
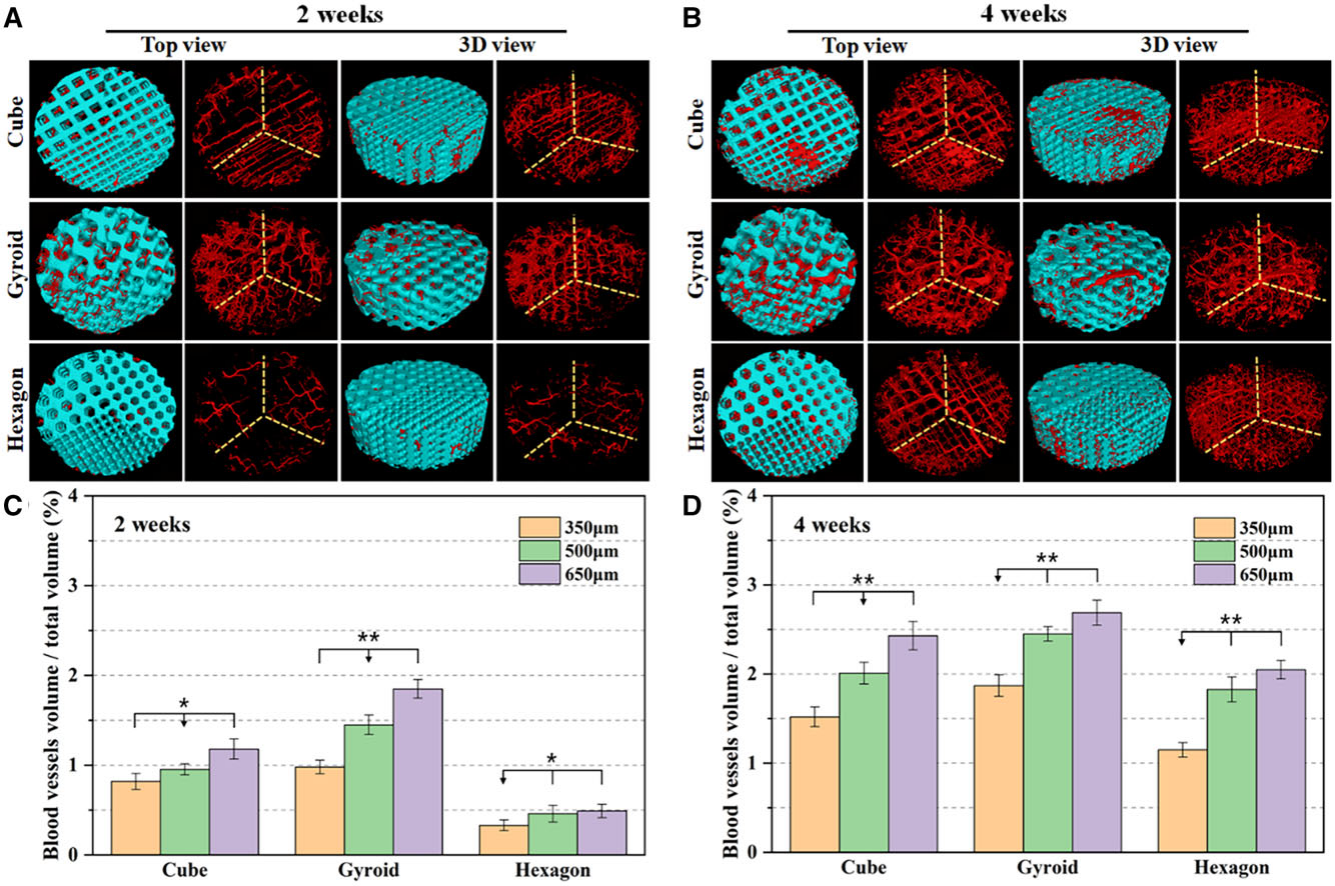
Representative Micro-CT top and 3D view images of micro-angiographic implants at 2 weeks (**A**) and 4 weeks (**B**) post-implantation. Blue: bioceramic scaffolds; red: blood vessels. Quantitative data of blood vessels BV/TV in the micro-angiographic implants at 2 weeks (**C**) and 4 weeks (**D**) postoperatively based on Micro-CT reconstruction analysis (*P < 0.05, **P < 0.01).

The μCT-reconstructed observation was used to evaluate the overall angiographic distribution and structural stability of bioceramic scaffolds. It indicated from Fig. 6A and B that the porous constructs of all of porous implants were retained well over time, and especially the integrated different pore dimensions were reconstructed after 4 weeks of implantation. The cube and hexagon pores could be easily distinguished, and the gyroid-pore scaffold showed three kinds of pore dimensions.

The 2D/3D μCT-reconstructed blood vessel networks (Fig. 6A and B) also exhibited some degree of relationship between angiogenesis and pore dimensions in a scaffold. It was very interesting that the gyroid pore showed appreciable angiogenesis after 2 weeks of implantation, though the 350- and 650-μm pore areas had significantly low and high density of blood vessels, respectively. However, the hexagon pores showed very limited new blood vessels after 2 weeks, regardless of the pore dimensions from 350 to 650 μm. As for the cube-pore scaffolds, the blood vessels could migrate into the small pores within a very short time stage (2 weeks), and neovessels were distributed homogeneously in the different regions with significantly different pore size. After 4 weeks, the angiogenesis was significantly enhanced in all three types of scaffolds. However, the gyroid- and cube-pore scaffolds showed larger blood vessels in the 500- and 650-μm pore regions. Meanwhile, the 350-μm pore region showed less blood vessels in the three types of scaffolds. Moreover, it could be observed that all of scaffolds retained their pore constructs throughout the whole experiment, without any structural collapse or crack, suggesting the appreciable structural stability of the CSi-Mg8 scaffolds in such dorsal muscle condition *in vivo*.

Additionally, the 3D μCT morphometric analysis was employed to quantify the new blood vessels in different pore regions. As has been verified by μCT reconstruction observation, quantitative parameters involving the BV/TV represented the relative density of blood vessels in the porous constructs. As shown in Fig. 6C and D, the gyroid-pore scaffolds exhibited significantly higher BV/TV ratio (≥1%) than the other two types of scaffolds after 2 weeks. Especially, the hexagon-pore scaffolds showed very low BV/TV ratio (<0.52%) in all of three pore dimension regions. With the prolongation of the implantation time up to 4 weeks, the BV/TV values of bioceramic scaffolds were increased by over 50%. The cube- and gyroid-pore scaffolds showed appreciable BV/TV ratio (>2.0%) in the 500- and 650-μm pore regions, but the hexagon-pore scaffolds showed BV/TV ratio of ∼1.8–2.0% in the 500- and 650-μm pore regions, which was nearly 3.5- to 4.0-fold higher than the data after 2 weeks. These quantitative results demonstrated that the curve pore geometry was more suitable for mediating angiogenesis in a very short time stage, and the strut-based pore geometries with lesser corners would be beneficial for blood vessel ingrowth.

### Histological analysis of vascularization

**Figure 7.**
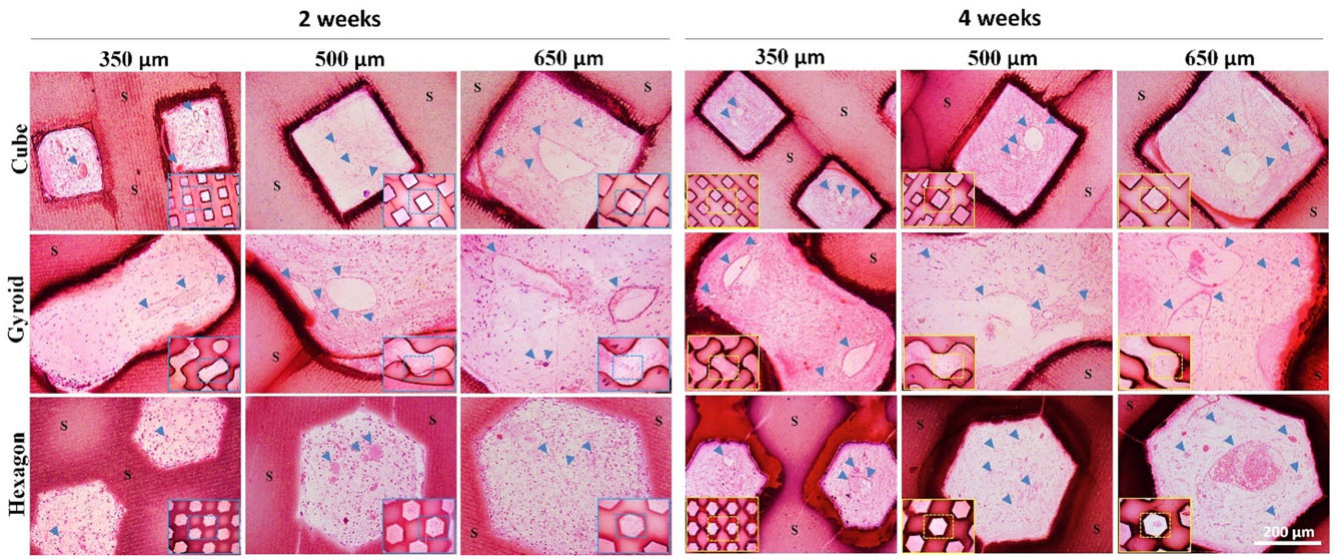
H&E staining images of sections of the bioceramic implants with integrated pore dimensions after implantation for 2 and 4 weeks, respectively. ‘S’ indicates the scaffolds and blood vessels are indicated by blue arrowheads.

Angiogenesis in the porous architectures of bioceramic scaffolds was characterized by H&E staining and was quantified by assessing the new blood vessel density and average of blood vessels. It can be observed from Fig. 7, the vascularization of porous scaffolds harvested at 2 and 4 weeks was revealed by H&E staining and the density of newly formed blood vessels in the gyroid- and cube-pore scaffold was higher than that in the hexagon-pore scaffolds. From the representative optical image of the transverse section of the scaffolds, it was obvious that the newly formed blood vessels readily grew into the randomly selected micropores of the scaffolds and even the red cells aggregated in the well-formed vessel in the large micropores after 4 weeks. Additionally, the bioceramic porous frameworks were observed clearly after 4 weeks, and in particular, the pore morphologies were maintained and could be easily distinguished among the three types of pore geometries.

**Figure 8.**
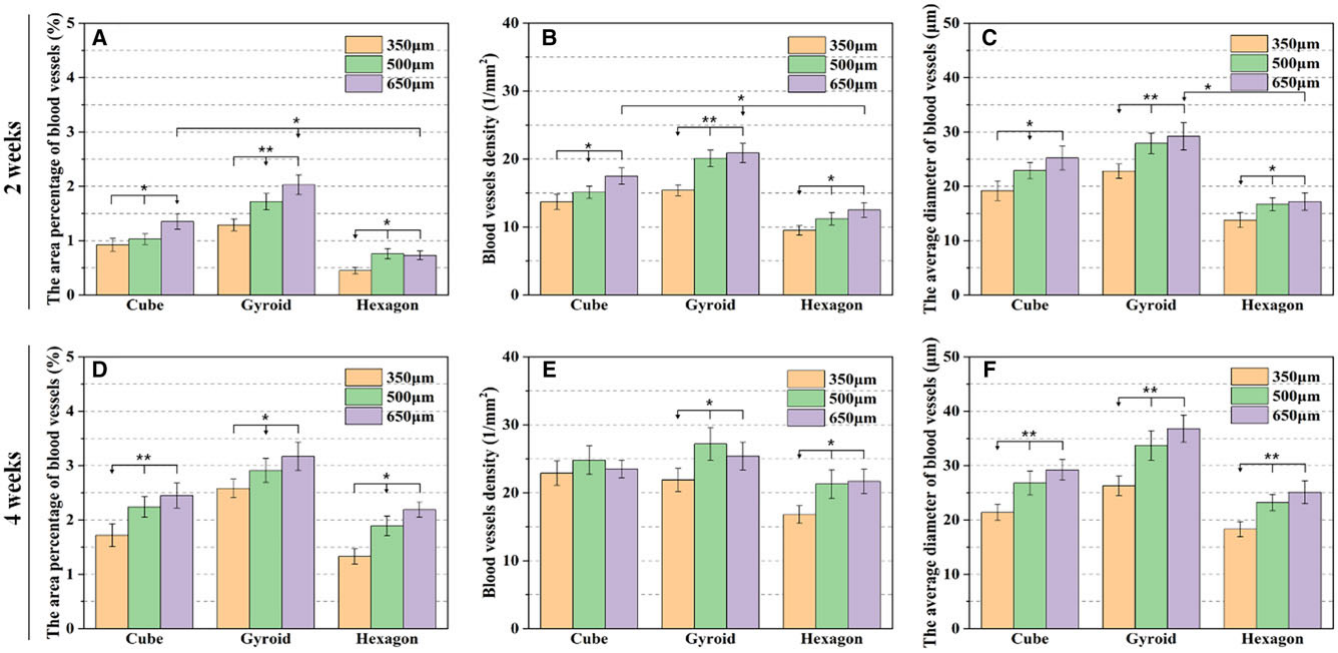
The area percentage of blood vessels (**A** and **D**), blood vessels density (**B** and **E**) and the average diameter of blood vessels (**C** and **F**) formed in the scaffolds with integrated pore dimensions at 2 and 4 weeks of post-implantation according to histomorphometric analysis (*P < 0.05, **P < 0.01).

According to the quantitative analysis as shown in Fig. 8, the area percentage of blood vessels, blood vessel density and the average diameter of blood vessels in the porous scaffolds were increased with time from 2 to 4 weeks. In particular, these data were increased totally with the pore dimension in a scaffold. Evidently, the blood vessel density in the hexagon-pore scaffolds was increased significantly from 2 to 4 weeks, while the average diameter of blood vessels in all of scaffolds were maintained relatively stably over time.

## Discussion

It is known that vascularization is one of the most important aspects for successfully regenerating the damaged bone tissue and always constitutes a major hurdle in bone repair medicine [4]. Bone tissues that have undergone significant damage or trauma exhibit an incredibly hypoxic environment due to the destruction of the local vascular network. Therefore, large bone defects that do not fully heal on their own are termed non-healing bone defects and exhibit a hypoxic condition [36]. Baino *et al.* [37] have also given a comprehensive overview of the current state-of-the-art and provided a picture for prospective research orbital implants and ocular prostheses. Polymeric and ceramic porous implants have gained prominence since their highly interconnected porous architecture allows them to act as a passive framework for fibrovascular ingrowth offering reduced complication rates. However, there are still drawbacks to these orbital implants including the risk of migration and extrusion, postoperative infections and low motility transmitted to the esthetic ocular prosthesis. Hence, the development of new biomaterials with enhanced functionalities (e.g. angiogenesis, *in situ* mouldability), which enable an improved outcome of eye replacement is more than ever desirable topics of research in the field of ocular implants [38].

In this study, the sintered CSi-Mg8 scaffolds with different pore architectures display large average pore size (>350 μm), high porosity (>55%) and appropriate compressive resistance (>15 MPa). In fact, the fabrication technique enables easy replication of the 3D model as making the printed scaffolds diversity of pore geometries. The mechanical properties are often related to the porosity and pore size, and appreciable strength is needed for the structure stability of bioceramic scaffolds during tissue reconstruction. Although the CSi-Mg8 bioceramic scaffolds display significantly different compressive strength due to the precise adjustment in the wall structure and size of unit cell, it is evident that there is a critical factor which may influence the mechanical properties of the macroporous bioceramics. In fact, the density of pore walls in the cube-typed unit cell is significantly increased, and these supporting pore walls would be inevitably helpful for enhancing the construct collapse. Based on the macroscopic and microscopic observations (Fig. 2), it demonstrates that the DLP-based 3D printing technique can completely replicate the finely pore geometry of 3D model with precisely tuned interior architecture and pore interconnectivity. Pore geometrical characteristic including porosity, pore size, pore interconnectivity and pore shape (internal architecture) significantly impact their mechanical properties and tissue regeneration capacity [23, 39]. In our study, the bioceramic scaffolds with similar porosity (55.1–56.5%) but different pore dimensions (350, 500 and 650 μm) show significantly different compressive strength and elastic modulus (Fig. 3). The unit cell of hexagon pore exhibited a more homogeneous stress distribution and lower stress maximum under compression load. Because of this structure feature, the hexagon-pore scaffolds showed higher mechanical resistance among three types of bioceramic scaffolds. Meanwhile, the gyroid-pore scaffolds have slightly higher specific surface area than the cube- and hexagon-pore scaffolds with similar pore dimensions. This may contribute on the faster ion release from the gyroid-pore scaffolds in the early stage.

In general, the early-stage nutrient transport is mainly carried out by diffusion processes that can supply the cells with nutrients only at a distance below 200 μm from the nearest capillary in the surrounding tissue, so that the cells in central area of pore construct frequently either die rapidly due to oxygen deficiency and lack of nutrient. Therefore, the architectural characteristics, such as pore size and interconnection of the biomaterials play an important role in revascularization of the scaffold. Bai *et al.* [20] reported that the pore parameters affect not only the size of the blood vessels growing into the porous structure but also the number of blood vessels formed in the pores of the bioceramic. They have found the increase in pore size only results in an increase in size of the blood vessels growing into the macroporous bioceramics, and with the increase in size of interconnection, both the size and number of the blood vessels formed in the macroporous increase. As for the closely packed microsphere-replica bioceramic scaffolds, there was no marked increase in extent of vascularization with further increase in pore size above 400 mm. Choi *et al*. [40] also developed the inverse opal scaffolds with uniform and precisely controlled pore diameters for systematically studying neovascularization *in vivo*. Their results reveal that scaffolds with small pores (79 and 147 μm) favor the formation of vascular networks with small vessels at high densities and poor penetration depth; by contrast, the scaffolds with large pores (∼224 and 312 μm) favor the formation of vascular networks with large blood vessels at low densities and deep penetration depth within 4 weeks.

However, in our study, the experimental results indicate that pore geometry is a more critical factor in influencing the early-stage vascularization efficiency. We found that the pore geometry is more important for vascularization in the bioceramic scaffold compared with the pore size. The pore size of hexagon-pore scaffolds is only slightly less than that in the gyroid-pore scaffolds, but the angiogenesis is significantly slow in the early stage. It is reasonable to consider that the surface pore size is of particular importance in affecting the cell migration and nutrient transport. On the other hand, the nominal pore size in the unit cell and the interconnected pore size (window size) are another two important parameters in influencing tissue ingrowth. In comparison with the microsphere-templated scaffold systems, the DLP-based scaffolds may readily fabricate porous constructs with appreciable pore size and pore interconnection. The conventional techniques used to prepare porous scaffolds include polymer foam replication [41], sacrificial templating [42] and phase separation method [43]. More recently, some direct or indirect 3D printing technologies [10, 44] offer more control over the porous architectures of scaffolds, providing a versatile tool for determining the effects of pore shape/architecture. However, the complex pore strut or curved pore surface is difficultly fabricated by the common directing ceramic ink writing [45]. In contrast, the DLP-based 3D printing is powerful in determining the effects of internal architectures and may manufacture a variety of complex pore architectures, which is independent on the limitation of shape of the nozzle in extrusion-based techniques. Thus, the pore size and pore interconnection could precisely tailored in the CSi-Mg8 bioceramic scaffolds with different pore geometries. Meanwhile, our studies could isolate the architectural parameters in 3D space, which is significantly helpful for understanding of the effects of pore geometry and pore size on the vascularization efficiency *in vivo*.

The 3D model analysis and SEM observation have confirmed the pore interconnection of the three types of bioceramic scaffolds is high enough for comparing the vascular cell migration and nutrient transport in the early stage. On the other hand, our studies may design the bioceramic scaffolds with different internal pore architectures while maintaining the similar porosity, pore size and surface area. Such design strategy is able to isolate the effects of pore geometry or pore size from other effects and achieve the precise determination of the effects of pore morphology in 3D scaffolds. Interestingly, the non-conventional gyroid-pore scaffold with curved pore surface showed appreciable angiogenesis within 2 weeks, suggesting the newly formed blood vessels have initiated quickly after implantation of the scaffolds. As unexpected, once the pore geometry is unfavorable for angiogenesis, much slower vascularization could be occurred in the larger pores in the hexagone-pore scaffolds. Accordingly, the rationale behind choosing porous biomaterial design is beyond a simple adjustment of pore architectures (pore size and interconnection), and the curvature (pore wall features) could simultaneously tailor the structural stability and bioactive ion release, and thus adjust vascularization or tissue reconstruction capability in the early stage.

## Conclusion

In summary, this study has designed porous bioceramic scaffolds with precisely tailored pore geometries and pore dimensions for *in situ* angiogenesis in the early stage *in vivo*. It has confirmed that although the gyroid-pore geometry exhibited limited compressive resistance, such pore architecture is beneficial for ion release and appreciable angiogenesis in the animal model in the early stage. However, the conventional cube or hexagon-pore architectures were only beneficial for enhancing mechanical strength but not early-stage vascularization. This study, for the first time, provide evidence that precisely tuned pore geometries in bioceramic scaffolds exhibit extremely different and intriguing angiogenesis responses in the early stage. These new findings suggest that the pore size and pore wall curvature design could not only mimic the trabecular architecture of biological minerals, but also need to optimize pore geometry beneficial for vascularization capability in the early stage. Based these concerns, the next work may be concentrated on the orbital reconstruction or bone regeneration efficacy of bioceramic scaffolds with curve- and strut-based pore topologies for translational medicine studies in the future.

## Supplementary data

Supplementary data are available at *REGBIO* online.

## Funding 

The authors would like to acknowledge the financial support from the National Key Research and Development Program of China (2017YFE0117700), the National Natural Science Foundation of China (81871775, 81902225 and 81772311), Zhejiang Provincial Natural Science Foundation of China (LBY21H060001, LGF21H060002 and Z22E029971), and the Medical and Health Research Project of Zhejiang Province (2020KY929 and 2020RC115). 

*Conflict of interest statement*. None declared. 

## Supplementary Material

rbab077_Supplementary_Data

## References

[1] Cheung WH , Miclau T, Chow SK-H, Yang FF, Alt V. Fracture healing in osteoporotic bone. Injury 2016;47:S21–6. 10.1016/S0020-1383(16)47004-X 27338222

[2] Bigham A , Foroughi F, Rezvani Ghomi E, Rafienia M, Neisiany RE, Ramakrishna S. The journey of multifunctional bone scaffolds fabricated from traditional toward modern techniques. Biodes Manuf 2020;3:281–306.

[3] Diomede F , Marconi GD, Fonticoli L, Pizzicanella J, Merciaro I, Bramanti P, Mazzon E, Trubiani O. Functional relationship between osteogenesis and angiogenesis in tissue regeneration. IJMS 2020;21:3242. 32375269 10.3390/ijms21093242 PMC7247346

[4] Saran U , Piperni SG, Chatterjee S. Role of angiogenesis in bone repair. Arch Biochem Biophys 2014;561:109–17. 25034215 10.1016/j.abb.2014.07.006

[5] LogithKumar R , KeshavNarayan A, Dhivya S, Chawla A, Saravanan S, Selvamurugan N. A review of chitosan and its derivatives in bone tissue engineering. Carbohydr Polym 2016;151:172–88. 27474556 10.1016/j.carbpol.2016.05.049

[6] Samavedi S , Whittington AR, Goldstein AS. Calcium phosphate ceramics in bone tissue engineering: a review of properties and their influence on cell behavior. Acta Biomater 2013;9:8037–45. 23791671 10.1016/j.actbio.2013.06.014

[7] Ni S , Lin K, Chang J, Chou L. Beta-CaSiO3/beta-Ca3(PO4)2 composite materials for hard tissue repair: in vitro studies. J Biomed Mater Res A 2008;85A:72–82. 10.1002/jbm.a.31390 17688291

[8] Kaur M , Singh K. Review on titanium and titanium based alloys as biomaterials for orthopaedic applications. Mater Sci Eng C Mater Biol Appl 2019;102:844–62. 31147056 10.1016/j.msec.2019.04.064

[9] Zhang L , Yang G, Johnson BN, Jia X. Three-dimensional (3D) printed scaffold and material selection for bone repair. Acta Biomater 2019;84:16–33. 30481607 10.1016/j.actbio.2018.11.039

[10] Marques A , Miranda G, Silva F, Pinto P, Carvalho O. Review on current limits and potentialities of technologies for biomedical ceramic scaffolds production. J Biomed Mater Res B Appl Biomater 2021;109:377–93. 32924277 10.1002/jbm.b.34706

[11] Barati D , Karaman O, Moeinzadeh S, Kader S, Jabbari E. Material and regenerative properties of an osteon-mimetic cortical bone-like scaffold. Regen Biomater 2019;6:89–98. 30967963 10.1093/rb/rbz008 PMC6446997

[12] Carrow JK , Di Luca A, Dolatshahi-Pirouz A, Moroni L, Gaharwar AK. 3D-printed bioactive scaffolds from nanosilicates and PEOT/PBT for bone tissue engineering. Regen Biomater 2019;6:29–37. 30740240 10.1093/rb/rby024 PMC6362822

[13] Wang J , Dai X, Peng Y, Liu M, Lu F, Yang X, Gou Z, Ye J. Digital light processing strength-strong ultra-thin bioceramic scaffolds for challengeable orbital bone regeneration and repair in Situ. Appl Mater Today 2021;22:100889.

[14] Wu C , Fan W, Zhou Y, Luo Y, Gelinsky M, Chang J, Xiao Y. 3D-printing of highly uniform CaSiO3 ceramic scaffolds: preparation, characterization and in vivo osteogenesis. J Mater Chem 2012;22:12288–95.

[15] Jiang S , Yu Z, Zhang L, Wang G, Dai X, Lian X, Yan Y, Zhang L, Wang Y, Li R, Zou H. Effects of different aperture-sized type I collagen/silk fibroin scaffolds on the proliferation and differentiation of human dental pulp cells. Regen Biomater 2021;8:rbab028. 34188954 10.1093/rb/rbab028 PMC8226109

[16] Wu R , Li Y, Shen M, Yang X, Zhang L, Ke X, Yang G, Gao C, Gou Z, Xu S. Bone tissue regeneration: the role of finely tuned pore architecture of bioactive scaffolds before clinical translation. Bioact Mater 2021;6:1242–54. 33210022 10.1016/j.bioactmat.2020.11.003 PMC7653208

[17] Xu M , Zhai D, Chang J, Wu C. In vitro assessment of three-dimensionally plotted nagelschmidtite bioceramic scaffolds with varied macropore morphologies. Acta Biomater 2014;10:463–76. 24071000 10.1016/j.actbio.2013.09.011

[18] Karageorgiou V , Kaplan D. Porosity of 3D biomaterial scaffolds and osteogenesis. Biomaterials 2005;26:5474–91. 15860204 10.1016/j.biomaterials.2005.02.002

[19] Jodati H , Yılmaz B, Evis Z. A review of bioceramic porous scaffolds for hard tissue applications: effects of structural features. Ceram Int 2020;46:15725–39.

[20] Bai F , Wang Z, Lu J, Liu J, Chen G, Lv R, Wang J, Lin K, Zhang J, Huang X. The correlation between the internal structure and vascularization of controllable porous bioceramic materials in vivo: a quantitative study. Tissue Eng Part A 2010;16:3791–803. 20673021 10.1089/ten.TEA.2010.0148

[21] Li J , Zhi W, Xu T, Shi F, Duan K, Wang J, Mu Y, Weng J. Ectopic osteogenesis and angiogenesis regulated by porous architecture of hydroxyapatite scaffolds with similar interconnecting structure in vivo. Regen Biomater 2016;3:285–97. 27699059 10.1093/rb/rbw031 PMC5043155

[22] Klenke FM , Liu Y, Yuan H, Hunziker EB, Siebenrock KA, Hofstetter W. Impact of pore size on the vascularization and osseointegration of ceramic bone substitutes in vivo. J Biomed Mater Res A 2008;85:777–86. 17896777 10.1002/jbm.a.31559

[23] Zadpoor AA. Bone tissue regeneration: the role of scaffold geometry. Biomater Sci 2015;3:231–45. 26218114 10.1039/c4bm00291a

[24] Lu F , Wu R, Shen M, Xie L, Liu M, Li Y, Xu S, Wan L, Yang X, Gao C, Gou Z. Rational design of bioceramic scaffolds with tuning pore geometry by stereolithography: microstructure evaluation and mechanical evolution. J Eur Ceram Soc 2021;41:1672–82.

[25] Melchels FP , Barradas AM, van Blitterswijk CA, de Boer J, Feijen J, Grijpma DW. Effects of the architecture of tissue engineering scaffolds on cell seeding and culturing. Acta Biomater 2010;6:4208–17. 20561602 10.1016/j.actbio.2010.06.012

[26] Babaie E , Bhaduri SB. Fabrication aspects of porous biomaterials in orthopedic applications: a review. ACS Biomater Sci Eng 2018;4:1–39. 33418675 10.1021/acsbiomaterials.7b00615

[27] Melchels FP , Bertoldi K, Gabbrielli R, Velders AH, Feijen J, Grijpma DW. Mathematically defined tissue engineering scaffold architectures prepared by stereolithography. Biomaterials 2010;31:6909–16. 20579724 10.1016/j.biomaterials.2010.05.068

[28] Baino F , Magnaterra G, Fiume E, Schiavi A, Tofan LP, Schwentenwein M, Verné E. Digital light processing stereolithography of hydroxyapatite scaffolds with bone-like architecture, permeability, and mechanical properties. J Am Ceram Soc 2021. DOI:10.1111/jace.17843.

[29] Xie J , Yang X, Shao H, Ye J, He Y, Fu J, Gao C, Gou Z. Simultaneous mechanical property and biodegradation improvement of wollastonite bioceramic through magnesium dilute doping. J Mech Behav Biomed Mater 2016;54:60–71. 26426432 10.1016/j.jmbbm.2015.09.012

[30] Yoo D. Heterogeneous minimal surface porous scaffold design using the distance field and radial basis functions. Med Eng Phys 2012;34:625–39. 22487098 10.1016/j.medengphy.2012.03.009

[31] Barba D , Alabort E, Reed RC. Synthetic bone: design by additive manufacturing. Acta Biomater 2019;97:637–56. 31394295 10.1016/j.actbio.2019.07.049

[32] He D , Zhuang C, Chen C, Xu S, Yang X, Yao C, Ye J, Gao C, Gou Z. Rational design and fabrication of porous calcium-magnesium silicate constructs that enhance angiogenesis and improve orbital implantation. ACS Biomater Sci Eng 2016;2:1519–27. 33440588 10.1021/acsbiomaterials.6b00282

[33] Kumagai H , Makihara T, Funayama T, Sato K, Noguchi H, Abe T, Koda M, Yamazaki M. Angiogenesis and new bone formation in novel unidirectional porous beta-tricalcium phosphate: a histological study. J Artif Organs 2019;22:294–9. 31325063 10.1007/s10047-019-01120-8

[34] Giannitelli SM , Accoto D, Trombetta M, Rainer A. Current trends in the design of scaffolds for computer-aided tissue engineering. Acta Biomater 2014;10:580–94. 24184176 10.1016/j.actbio.2013.10.024

[35] Ahmadi SM , Yavari SA, Wauthle R, Pouran B, Schrooten J, Weinans H, Zadpoor AA. Additively manufactured open-cell porous biomaterials made from six different space-filling unit cells: the mechanical and morphological properties. Materials (Basel) 2015;8:1871–96. 28788037 10.3390/ma8041871 PMC5507048

[36] Glowacki J. Angiogenesis in fracture repair. Clin Orthop Relat Res 1998;355:S82–9. 10.1097/00003086-199810001-00010 9917629

[37] Baino F , Perero S, Ferraris S, Miola M, Balagna C, Verne E, Vitale-Brovarone C, Coggiola A, Dolcino D, Ferraris M. Biomaterials for orbital implants and ocular prostheses: overview and future prospects. Acta Biomater 2014;10:1064–87. 24342039 10.1016/j.actbio.2013.12.014

[38] Baino F , Potestio I. Orbital implants: state-of-the-art review with emphasis on biomaterials and recent advances. Mater Sci Eng C Mater Biol Appl 2016;69:1410–28. 27612842 10.1016/j.msec.2016.08.003

[39] Jones AC , Arns CH, Hutmacher DW, Milthorpe BK, Sheppard AP, Knackstedt MA. The correlation of pore morphology, interconnectivity and physical properties of 3D ceramic scaffolds with bone ingrowth. Biomaterials 2009;30:1440–51. 19091398 10.1016/j.biomaterials.2008.10.056

[40] Choi SW , Zhang Y, Macewan MR, Xia Y. Neovascularization in biodegradable inverse opal scaffolds with uniform and precisely controlled pore sizes. Adv Healthc Mater 2013;2:145–54. 23184495 10.1002/adhm.201200106 PMC3541475

[41] Ji C , Annabi N, Hosseinkhani M, Sivaloganathan S, Dehghani F. Fabrication of poly-DL-lactide/polyethylene glycol scaffolds using the gas foaming technique. Acta Biomater 2012;8:570–8. 21996623 10.1016/j.actbio.2011.09.028

[42] Descamps M , Duhoo T, Monchau F, Lu J, Hardouin P, Hornez JC, Leriche A. Manufacture of macroporous β-tricalcium phosphate bioceramics. J Eur Ceram Soc 2008;28:149–57.

[43] Goh YQ , Ooi CP. Fabrication and characterization of porous poly(L-lactide) scaffolds using solid-liquid phase separation. J Mater Sci Mater Med 2008;19:2445–52. 18219558 10.1007/s10856-008-3366-9

[44] Hollister SJ. Porous scaffold design for tissue engineering. Nat Mater 2005;4:518–24. 16003400 10.1038/nmat1421

[45] Peng E , Zhang D, Ding J. Ceramic robocasting: recent achievements, potential, and future developments. Adv Mater 2018;30:e1802404. 30306642 10.1002/adma.201802404

