# Supplementary figures and images for: Integrating pore architectures to evaluate vascularization efficacy in silicate-based bioceramic scaffolds
Source: Regen Biomater. 2021 Dec 16;9:rbab077. doi: 10.1093/rb/rbab077 (PMC9039507; doi:10.1093/rb/rbab077)

**Supplementary data**


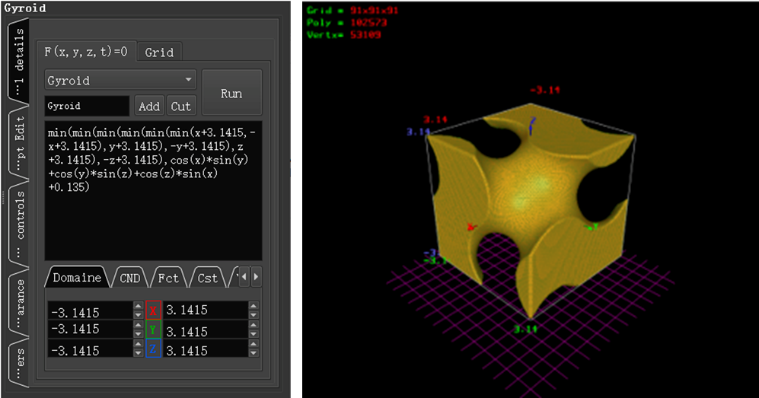


Figure S1. The design of the unit cell model of gyroid by mathmod.

Supplement: rbab077_Supplementary_Data [file rbab077_supplementary_data.docx]
